# Mercury, Fatty Acids Content and Lipid Quality Indexes in Muscles of Freshwater and Marine Fish on the Polish Market. Risk Assessment of Fish Consumption

**DOI:** 10.3390/ijerph14101120

**Published:** 2017-09-25

**Authors:** Joanna Łuczyńska, Beata Paszczyk, Joanna Nowosad, Marek Jan Łuczyński

**Affiliations:** 1 Chair of Commodity and Food Analysis, University of Warmia and Mazury in Olsztyn, ul. Plac Cieszyński 1, 10-726 Olsztyn, Poland; paszczyk@uwm.edu.pl; 2 Department of Lake and River Fisheries, University of Warmia and Mazury in Olsztyn, 10-726 Olsztyn, Poland; nowosad.joanna@gamil.com; 3 Department of Ichthyology, The Stanisław Sakowicz Inland Fisheries Institute in Olsztyn, 10-719 Olsztyn, Poland; marekjl@infish.com.pl

**Keywords:** fish, mercury, fatty acids, quality index, human health, EWI, THQ

## Abstract

Mercury content and fatty acids in muscles of *Perca fluviatilis* L. (European perch), *Leuciscus idus* L. (ide), *Cyprinus carpio* L. (European or common carp), *Oncorhynchus mykiss* Walb. (rainbow trout), *Platichthys flesus* L. (European flounder). and *Clupea harengus* L. (bream) from the Polish market were investigated. The total mercury was processed with AAS. The fatty acids were analyzed by gas chromatography. The concentration of mercury in muscles varied from 0.006 to 0.138 mg/kg and decreased as follows: perch ≈ ide > flounder > herring ≈ bream ≈ rainbow trout > carp (*p* ≤ 0.05). There were only significant positive correlations between body weight and mercury content in muscle tissue of carp (r = 0.878), flounder (r = 0.925) and herring (r = 0.982) (*p* ≤ 0.05). The atherogenic index (AI), thrombogenicity index (TI) and flesh-lipid quality index (FLQ) were calculated as follows 0.33–0.70 (IA), 0.16–0.31 (IT) and 13.01–33.22 (FLQ). Hypocholesterolemic (OFA) and hypercholesterolemic fatty acids (DFA) in muscles of fish ranged from 18.26 to 23.01 and from 73.91 to 78.46, respectively. In most cases, there were not significant correlations between size (body weight and total length) and fatty acids in the muscles of the examined fish (*p* > 0.05). The Target Hazard Quotient (THQ) values were below 1, which shows that there is no non-carcinogenic health risk to the consumer by consuming the examined fish.

## 1. Introduction

Fish are an important sources of biologically valuable proteins, fats, fat-soluble vitamins and n-3 polyunsaturated fatty acids with five and six double bonds in the carbon chain [1]. The results of prospective cohort studies indicate that consuming fish or fish oil containing the n-3 fatty acids eicosapentaenoic acid (EPA) and docosahexaenoic acid (DHA) is associated with decreased cardiovascular death risk, whereas the consumption of vegetable oil–derived n-3 fatty acid α-linolenic acid is not as effective [2]. Long-chain fatty acids can be classified into n-3 fatty acids and n-6 polyunsaturated fatty acids which are beneficial to health, because they have anti-inflammatory properties and decrease the risk of cardiovascular disease, cancer, hypotriglyceridemia and increase HDL cholesterol [3,4,5,6,7]. They also (along with nutrients such as carotenoids, vitamins A, D, E, C and zinc, selenium and iron) influence immune system activity [8]. Saturated fatty acids such as lauric (C12:0), myristic (C14:0) and palmitic (C16:0) acid increase total and LDL-cholesterol concentrations [9,10]. Stearic acid (C18:0) is neutral or hypocholesterolemic [11,12,13]. Polyunsaturated fatty acids elicit the most potent hypocholesterolemic effects [14].

In contrast, fish are also vulnerable to some chemical pollutants. It is well known that fish are also an important ecological link in the food chain. They serve as food for other fish, wildlife, and humans and they are indicators of water quality and ecosystem health, because they alert people to changes in water quality [15]. The content of heavy metals, including mercury, discovered in some fish makes it difficult to clearly establish the role of fish consumption on a healthy diet [16], particularly since certain elements, such as mercury (present in organisms of lower trophic levels) can be efficiently transferred to higher-level organisms, becoming more concentrated at the top of the food chain [17]. Human exposure to mercury may occur via a variety of pathways, including consumption of fish, occupational and household uses, dental amalgams and mercury-containing vaccines [18].

Mercury usually combines with other elements to form various inorganic (e.g., the mineral cinnabar, a combination of mercury and sulfur) and organic (e.g., methylmercury-MeHg) compounds, although Hg occasionally also occurs in its elemental, relatively pure form, as a liquid or vapor [18]. Elemental mercury is lipid soluble and can cross the blood-brain barrier, while inorganic mercury compounds are not lipid soluble, rendering them unable to cross the blood-brain barrier [19]. The high exposure to mercury induces changes in the central nervous system, behavioral changes, tremors, headaches, hearing and cognitive loss, dysarthria, incoordination, hallucinations and death, whereas in the cardiovascular system, this metal induces hypertension in humans and animals [20]. The most dangerous form of mercury is methylmercury. The developing human brain is particularly susceptible to injury caused by MeHg, which easily passes the placental barrier [21]. According to these authors methylmercury can have serious adverse effects on the developing nervous system and may promote heart diseases. This form of mercury is bioaccumulated to a high degree in aquatic food chains to attain its highest concentrations in edible tissues in long-lived predatory fish living in both fresh and ocean waters [22]. 

In fish, the contribution of methylmercury to total mercury generally ranges between 30% and 100%, depending on species of fish, size, age and diet. The Panel on Contaminants in the Food Chain (CONTAM) Panel used a conservative approach to calculate methylmercury dietary exposure by assuming that 100% of mercury in fish is in the form of methylmercury [23]. According this CONTAM Panel, in order to ensure that dietary exposure to inorganic mercury was not underestimated, 20% of total mercury in fish was simultaneously assumed to be inorganic mercury when calculating inorganic mercury dietary exposure. There are fish species that have low in Hg (i.e., <0.1 mg/kg) and rich sources of n-3 polyunsaturated fatty acids PUFA. In contrast, other fish species are both highly contaminated with Hg (~1 mg/kg and higher) and are not especially rich sources of n-3 PUFA [24]. The maximum residue level (MRL) of Hg recommended by EU for fish is 0.5 mg/kg wet weight and 1 mg/kg in predatory fish such as shark, swordfish, tuna and pike. Domingo [25] said that adequately balancing adequately the risks and benefits of fish consumption is currently a key nutritional/environmental health issue. Essential aspects in balancing the benefits and risks of regular consumption include the choice of the most suitable fish species and their size (both in terms of PUFA and pollutants, as well as the frequency and amount consumed and the way in which it is served [13,25]. The Tolerable Weekly Intake (TWI) for inorganic mercury is expressed as mercury is 4 μg/kg body weight, whereas TWI for methylmercury is expressed as mercury is 1.3 μg/kg body weight [23], whereas the Adequate Intake of 250 mg for eicosapentaenoic acid plus docosahexaenoic acid for adults based on cardiovascular considerations [10]. The objective of this study was to determine the mercury content, profile of fatty acids and the lipid quality indexes (atherogenic index (AI), thrombogenicity index (TI) and flesh-lipid quality index (FLQ) in muscles of important freshwater and marine fish species from the Polish market. Additionally, this study sought to evaluate the dependence between biometric parameters (body weight and total length) and mercury and fatty acids content. The health risk assessment with mercury was determined by using THQ.

## 2. Materials and Methods

### 2.1. Sample Preparation

Perch (*Perca fluviatilis* L.), ide (*Leuciscus idus* L.), carp (*Cyprinus carpio* L.), rainbow trout (*Oncorhynchus mykiss* Walb.), flounder (*Platichthys flesus* L.) and herring (*Clupea harengus* L.) were bought on the Polish market. The samples of fish were collected the same day. The body weight and total length were measured (Table 1). Muscles (without skin) were dissected from the dorsal part and stored until analysis in the refrigerator at −18 °C. For large bream, perch, ide, carp and rainbow trout, the samples were prepared from muscles taken from one specimens, whereas for flounder and herring, samples were prepared from muscle tissue taken from two fish. 

Ethical statement: according to European and Polish Law, the research done on the commercially catch fishes tissue is free to obtain permission on Local Ethical Commision.

### 2.2. Analytical Methods

#### 2.2.1. Mercury

Duplicate samples of muscles were weighed into quartz boats (270 mg ± 0.0001 g) and transferred from an analytical auto-sampler. The total mercury was processed with atomic absorption spectrometry thermal decomposition using a Milestone DMA-80 (with dual-cell) instrument (Milestone, Sorisole, Italy). The samples are first dried at 160 °C by 60 s and then underwent decomposition in a furnace in an oxygen atmosphere (temp. 650 °C by 60 s). The time between the end of drying and the beginning of decomposition (650 °C) is 120 s. The absorption wavelength was 253.65 nm (detection limit—0.005 ng Hg) and detector was a UV enhanced photodiode instrument. The analysis method was tested by measuring the elements in reference material: BCR CRM 422 (muscles of cod *Gadus morhua* (L.)) with a certified mercury value. The recovery rate percentage was 100.2% (*n* = 4).

#### 2.2.2. Fat and Fatty Acids Analysis

Approximately 1 g samples (±0.0001 g) in duplicate were dried to a constant weight at 105 °C in glass sample tubes with frits and transferred to weighed beakers. The lipids from the fish muscles (without skin) and liver were extracted according to the hot extraction method using an E-816HE automatic extractor. The analysis consisted of three steps (extraction, rinsing, drying). After the extraction was finished, all of the solvent (petroleum ether) was collected in the tank. Fat was dried in beaker at 100 °C to a constant weight and was then weighed. 

The content of fat (%) was calculated according to pattern: x = [(b − a) × 100]/c, where: a = weight of flask (g), b = weight of flask with extracted fat (g), c = weight of samples (g).

The lipids were extracted according to the Folch’s procedure [26]. The studied material was broken up and mixed. 2 g of sample was homogenised for 1 min with 20 mL of methanol. Next, 40 mL chloroform was added and the procedure was continued for 2 min. The prepared mixture was filtered to a 250 mL glass cylinder. The solid residue was re-suspended in 60 mL chloroform: methanol (2:1 *v*/*v*) and homogenized again for 3 min. After filtering, the solid was washed once more with 40 mL chloroform and once with 20 mL methanol. The combined filtrate was transferred to the same cylinder. 0.88% sodium chloride in water (determining 1/4 volume of filtrate) was added to the total filtrate and then shaken and left overnight. The upper layer was removed and to the lower layer a water:methanol mixture (1:1 *v*/*v*) was added and the washing procedure was repeated. The remaining layer was trickled by anhydrous sodium sulphate and distilled by means of aggregate for distillation of solvents. The fatty acid methyl esters were prepared from total lipids with the Peisker method with chloroform:methanol:sulphuric acid (100:100:1 *v*/*v*) [27].

The fatty acids of methyl esters of each sample were analyzed using a 7890A chromatograph (Agilent Technologies, Waldbroon, Germany) equipped with a flame-ionization detector (FID) under the following conditions: capillary column (dimension 30 m × 0.25 μm with a 0.32 mm internal diameter, liquid phase Supelcowax 10 (Supelco, Bellefonte, PA, USA), temperature: flame-ionization detector −250 °C, injector −230 °C, column −195 °C, carrier gas—helium with a flow rate 1.5 mL/min. Individual fatty acids were identified by comparing the relative retention time peaks to the known Supelco standards. 

### 2.3. The Llipid Quality Indexes Were Calculated from the Fatty Acids Composition Using the Following Formulae

#### 2.3.1. Index of Atherogenicity (AI) 

The AI indicates the relationship between the sum of the main saturated fatty acids and that of the main classes of unsaturated fatty acids, the former being considered proatherogenic (favoring the adhesion of lipids to cells of the immunological and circulatory system), and the latter antiatherogenic (inhibiting the aggregation of plaque and diminishing the levels of esterified fatty acid, cholesterol, and phospholipids, thereby preventing the appearance of micro and macro coronary diseases) [28,29,30]

(AI) = [C12:0 + (4 × C14:0) + C16:0]/(n-3PUFA + n-6PUFA + MUFA)

PUFA—polyunsaturated fatty acids
MUFA—monounsaturated fatty acids
C12:0—lauric acid, C14:0—myristic, C16:0—palmitic.

#### 2.3.2. Index of Thrombogenicity (TI) 

The TI shows the tendency to form clots in the blood vessels. This is defined as the relationship between the prothrombogenetic (saturated) and the antithrombogenetic fatty acids (MUFA, n-6 PUFA and n-3PUFA) [28,29,30]:

(TI) = [C14:0 + C16:0 + C18:0]/[(0.5 × C18:1) + (0.5 × sum of other MUFA) + (0.5 × n-6PUFA) + (3 × n-3PUFA) + n-3PUFA/n-6PUFA)]

#### 2.3.3. Flesh-Lipid Quality (FLQ) 

The FLQ indicates the percentage correlation between the main n-3 PUFA (EPA + DHA) and the total lipids. The higher value of this index is an indicator of the higher quality of the dietary lipid source [31,32]:

(FLQ) = 100 × [EPA + DHA]/[% of total fatty acids]
Hypocholesterolemic fatty acids (OFA): (OFA) = C12:0 + C14:0 + C16:0
Hypercholesterolemic fatty acids (DFA): (DFA) = C18:0 + UFA
EPA—eicosapentaenoic acid (C20:5)
DHA—docosahexaenoic (C22:6)
UFA—unsaturated fatty acids (MUFA + PUFA)
C18:0—stearic acid

### 2.4. Human Health Risk Assessment

#### 2.4.1. Estimated Daily Intake of Heavy Metals

EDI—the estimated daily intake (μg/kg body weight/day) = C × IR/BW
TWI—Tolerable Weekly Intake = EDI × 7
C—the average concentration of heavy metals in food stuffs (μg/g wet weight)
IR—the daily ingestion rate (g/daily)
The fish consumption was 12.1 kg per capita/year [33]
BW—the average body weight (60 kg) [34]

#### 2.4.2. Target Hazard Quotient (THQ)

The THQ assesses the non-carcinogenic health risk of consumers due to the intake of heavy metal polluted fish using the oral reference dose (RfD = 3.00 × 10^−4^) [35,36]. The non-cancer risk model is used in this study because mercury is not classifiable as a human carcinogen. When THQ < 1 there is health benefit from fish consumption and the consumer is safe, whereas THQ > 1 suggests a high probability of adverse human health risks:

THQ = (EFr × ED × FiR × C/RfD × BW × TA) × 10^−3^
Efr—the Exposure Frequency (365 days/year)
ED—the Exposure Duration (70 years)
FiR—the Fish Ingestion Rate (g/person/day)
C—the average concentration of heavy metals in food stuffs (μg/g wet weight)
RfD—the oral reference dose (mg/kg/day) (USEPA 2017)
BW—the average body weight of local residents (60 kg) [34]
TA—is the average exposure time (365 days/year × ED)

#### 2.4.3. Statistical Analysis

Significant interspecific differences in the content of fatty acids and mercury in the muscles were calculated using a one-way analysis of variance ANOVA (Duncan’s test) after testing for homogeneity of variance (test Levene’s). Differences were found to be significant at *p* ≤ 0.05. The correlation coefficients between the content of Hg and fatty acids in muscles of fish were calculated using the STATISTICA 10 software (StatSoft, Kraków, Poland). Similarly, the correlation coefficients between the content of Hg and fatty acids in muscle tissue of fish and their size (body weight and total length) were evaluated using the STATISTICA 10 software. The significance level of *p* ≤ 0.05 was used.

## 3. Results

The content of total lipid varied widely within and among species (Table 1). Significantly higher values of total lipid were observed in muscles of herring (*p* ≤ 0.05). The content of the element in the muscles of the examined fish was as follows: herring (11.49%) > rainbow trout (2.05%) ≈ flounder (1.77%) and flounder ≈ ide (0.80%) and ide ≈ carp (0.68%) ≈ perch (0.35%) ≈ bream (0.12%).

Muscles of bream were characterized by a significantly higher content of saturated fatty acids SFA (32.94%) (*p* ≤ 0.05), whereas the content of monounsaturated fatty acids MUFA (54.54%) was significantly higher in muscle tissue of herring (*p* ≤ 0.05) than in the other fish examined (Table 2). In the case of bream and perch as representative wild freshwater fish, the muscles of these fish contained significantly more n-3 PUFA and n-3 HUFA than marine fish (flounder and herring), cultured fish (carp and rainbow trout) and ide inhabiting different aquatic ecosystems (river and lakes) (*p* ≤ 0.05). The values n-3 PUFA in muscle tissue of bream and perch were 37.46% and 38.62%, while the contents of n-3 HUFA were 35.00% and 36.57%, respectively. However, the muscles of bream, carp and rainbow trout had significantly higher content of n-6 PUFA (15.48%, 15.10% and 15.03%, respectively).

A significantly lower amount of hypocholesterolemic fatty acids was observed in muscles of rainbow trout (18.26%) and flounder (20.34%) (*p* ≤ 0.05) than other fish examined, although there were no significant differences between muscle tissue flounder and carp, ide, and bream (*p* > 0.05) (Table 2). hypercholesterolemic fatty acids contents in the muscles of fish were as follows: flounder (78.46%) ≈ carp (77.55%) ≈ ide (77.33%) and carp ≈ ide ≈ herring (76.07%) ≈ perch (75.73%) and herring ≈ perch ≈ bream (74.88%) ≈ rainbow trout (73.91%). The muscles of herring had significantly higher index of atherogenicity (0.70) than other fish studied (*p* ≤ 0.05), whereas the muscle tissue of carp had a significantly higher index of thrombogenicity (0.31) (*p* ≤ 0.05). There were also significant differences between the value of flesh-lipid quality in perch (33.22) and other fish examined (*p* ≤ 0.05).

Muscle tissue of perch and ide contained more mercury (0.139 and 0.109 mg/kg, respectively) than other fish studied (*p* ≤ 0.05) (Table 1), while a representative marine fish such as flounder had more mercury than herring (0.021 mg/kg), bream (0.016 mg/kg), rainbow trout (0.015 mg/kg) and carp (0.006 mg/kg) (*p* ≤ 0.05). The differences in the content of mercury in muscles of herring, bream and rainbow trout were not significant (*p* > 0.05). However, the muscles of carp contained a significantly lower mercury concentration than other fish examined (*p* ≤ 0.05). The mercury content in muscles of the examined fish did not exceed maximum residue level (0.5 mg/kg).

Positive correlation coefficients were found between mercury levels in the muscles and the fish weight and length (Table 1). However, significant correlations were found between body weight and the content of mercury in muscle tissue of carp (r = 0.878, *p* = 0.050), flounder (r = 0.925, *p* = 0.008) and herring (r = 0.982, *p* = 0.0005).

Negative correlations were noted between the total length and the content of C18:2 (n-6) in muscles of bream (r = −0.845, *p* = 0.034), as well as between the total length and AI (r = −0.890, *p* = 0.018) or TI (r = −0.812, *p* = 0.050) in muscles of ide (Table 3). The correlation coefficients between length and ƩPUFA (r = 0.835, *p* = 0.038), Ʃn-3 PUFA (r = 0.821, *p* = 0.045), Ʃn-3 HUFA (r = 0.836, *p* = 0.038) or DFA (r = 0.837, *p* = 0.038) in muscles of ide were significantly positive. There was also a positive correlation between length and C18:2(n-6) (r = 0.927, *p* = 0.024) in muscles of perch and the ratio n-3/n-6 in muscle tissue of bream (r = 0.876, *p* = 0.022). The content of C18:2(n-6) in muscles of perch and carp was positively correlated with body weight (r = 0.908, *p* = 0.033 and r = 0.883, *p* = 0.047, respectively). Similarly, a positive correlation was observed between C20:5(n-3) in bream (r = 0.816, *p* = 0.047), C14:0 in flounder (r = 0.888, *p* = 0.018) or ƩMUFA in rainbow trout (r = 0.835, *p* = 0.039) and the body weight of these fish. However, there were negative correlation coefficients between body weight and C20:5(n-3), C20:5(n-3) in muscle tissue of perch of r = −0.930, *p* = 0.022 and r = −0.916, *p* = 0.029, respectively as well as ƩPUFA, Ʃn-3 PUFA, Ʃn-3 HUFA and FLQ in muscles of rainbow trout (r = −0.887, *p* = 0.018, r = −0.845, *p* = 0.034, r = −0.831, *p* = 0.040 and r = −0.830, *p* = 0.041, respectively).

### Human Health Risk Assessment

The THQ for mercury in different fish species is presented in Table 4. THQ values were below 1 which shows that there is no non-carcinogenic health risk to the consumer by consuming the examined fish. The EDI of mercury from the 33.16 g portions of fish was: 0.009 μg/body weight (bream), 0.076 (perch), 0.060 (ide), 0.003 (carp), 0.008 (rainbow trout), 0.031 (flounder) and 0.012 μg/body weight (herring). The weekly intake of mercury (232.12 g of fish portion) accounts for 1.50, 13.34, 10.57, 0.60, 1.42, 5.45, 2.03% of the TWI (as 4 μg/kg body weight) and 4.611, 41.056, 32.527, 1.845, 4.363, 16.760 and 6.248% of the TWI (as 1.3 μg/kg body weight).

## 4. Discussion

Previously findings reported by Łuczyńska and Krupowski [37] showed that mercury content in muscles of fish from the Polish market varied between some species. The muscles of predatory (perch and pike) and non-predatory freshwater fish (bream) contained higher levels of mercury than marine fish (flounder and mackerel) (*p* ≤ 0.05). Similarly, predatory freshwater fish (i.e., pike) had more mercury than non-predatory fish (i.e., bream). For bream and flounder, a contrary regularity was found (Table 1). Muscle tissue perch from the Vistula River (Toruń, Poland) contained more mercury (0.36 mg/kg) than muscles of bream (0.054 mg/kg) [38]. Muscles tissue of flounder contained a higher concentration of mercury (0.036 mg/kg) than muscles of herring (0.032 mg/kg) [39]. Voigt [40] observed differences between the content of mercury in the muscles of the pelagic open-sea species (herring) and inshore species (perch) (Western Estonia). According to the same author perch contained more mercury than herring. The observations of above authors are close to those found in the present study. Kenšová et al. [41] also found the highest concentration of mercury in muscles of predatory fish (asp *Aspius aspius* L., eel *Anguilla anguilla* L., pike *Esox lucius* L., and perch). The same authors showed that among non-predatory fish (carp, bream, tench *Tinca tinca* L. and roach *Rutilus rutilus* L.) the lowest mercury content were noted in carp. Zrnčić et al. [42] studied 14 different fish species belonging to four groups according to feeding habits (among others: ide, carp and bream). The authors found that the differences between the content of metals examined, including mercury, in the four groups (herbavore, omnivore, piscivore and plankton-feeding fish) were significant. These results are consistent with those in present study (Table 1). Popov et al. reported that interspecies differences in the content of metals, including mercury, are most likely caused by peculiarities in the feeding habits of fish (Russia) [43]. According to those authors, the contents of mercury in muscles of perch, bream and ide were 0.033, 0.035 and 0.014 mg/kg. There were statistically significant differences between content of mercury in muscles of herring (0.0658 mg/kg) and carp (0.0373 mg/kg) bought in Polish market (*p* < 0.05) [44]. Muscle tissue of carp from the Neretva River (Croatia) had higher values of mercury (0.190 mg/kg) than carp from the Polish market studied by the above authors [45]. The fish (carp and rainbow trout (Poland)) contained 0.036 mg Hg/kg [46]. According to Mazej et al. [47] the muscles of perch from Velenjsko (Slovenia) had four times higher content than muscle tissue of carp (0.03 mg/kg). Lidwin-Każmierkiewicz et al. [48] found that muscle tissue of perch from West Pomerania (Poland) contained more mercury (0.03 mg/kg) than carp and bream (0.01 mg/kg). Those authors did not observe differences between the mercury concentration in muscles of carp and bream. However, Kenšová et al. [49] noted a significant differences between the content of mercury in muscles of carp and predatory fish species (asp *Aspius aspius* L., pike *Esox Lucius* L. and pikeperch *Sander lucioperca* L.) caught in the Vĕstonice Reservoir (Czech Republic) (*p* < 0.01), but did not find significant differences between the mercury concentration in muscle tissue of carp and bream (*p* > 0.01) or any dependence of metal content on fish weight, age or sex. For the muscle tissue of fish from Puck Buy, perch (0.110–0.130 mg/kg) contained higher values of mercury than other fish, i.e., bream (0.040 mg/kg) flounder (0.031–0.053 mg/kg) and herring (0.049 mg/kg) [50]. The same authors found a positive relationship (*p* < 0.05) only between total body length and weight and mercury content in muscles of flounder. Baeyens et al. [51] found strong positive correlation between the length of flounder and the concentration of mercury (r = 0.71). This is in accordance with the results of the present study, but only in relation to correlation of mercury content in muscles of flounder with body weight.

The content of mercury in muscle of perch from Lake Gusinoye and the Selenga River (Russia) significantly depended on the fish length and weight (r = 0.62–0.90, *p* < 0.01) [52]. According to Łuczyńska [53], the positive correlation between the body weight and the total mercury levels in muscles of perch from Lake Łańskie, Pluszne, Dłużek and Maróz (r = 0.967, 0.963, 0.876 and 0.967, *p* < 0.001, respectively) was slightly higher than that between mercury and body length (r = 0.933, 0.950, 0.781 and 0.916, *p* < 0.001), respectively). A positive correlation between the concentration of mercury in muscles of perch from the southern Baltic and weight or length (*p* < 0.01) was found by Szefer et al. [54]. Mercury content in muscles of predatory fish belonging to five species (asp, *Aspius aspius* L.; eel *Anquilla anquilla* L.; perch; pike, *Esox Lucius* L.; pikeperch, *Stizostedion lucioperca* L.) from the Želivka Reservoir was correlated with weight (r = 0.330, *p* < 0.001) [55]. The above findings were not confirmed by the results of this studyof perch.

There were also significant positive correlation between mercury content and fish body size (ide, carp and bream) [42]. A significant correlation coefficient between the concentration of mercury in muscle tissue of bream from Lake Balaton and their length (r = 0.8459, *p* < 0.0001) was also observed by Farkas et al. [56]. This is in accordance with the examined results, but only in the case of carp.

According to Łuczyńska et al. [57], the content of fat and fatty acids varied both between and within species. The muscles of bream and perch contained 1.03 and 0.89 of total lipid, respectively. The fat content in fillets of carp and bream from Inland waters was 3.24% and 7.13%, respectively [58]. These results are higher to those for the fish examined (Table 1). The muscle tissue of rainbow trout studied had lower content of fat than meat of rainbow trout from extensive farming (3.13%) and intensive farming (5.39%) [59] and fish of the same species from Polish market (6.84%) [60]. Polak-Juszczak and Adamczyk [61] found that muscles of bream, perch and herring contained 3.14%, 0.12% and 2.61% of fat. This literature data was not confirmed the presented findings. 

Ljubojevic et al. [58] found differences between the content of MUFA, PUFA, n-3 PUFA and n-6 PUFA in muscles of bream and carp (*p* < 0.01). According to these authors fillets of bream contained more MUFA and n-3 PUFA than carp fillets, and lower amounts of PUFA and n-6 PUFA. These results are close to those for MUFA and n-3 PUFA in muscles of the fish studied (Table 2). Polak-Juszczak and Komar-Szymczak [62] studied the fatty acids profiles in muscles of bream, perch and herring from the Vistula Lagoon (Poland). They authors found that the content of SFA, MUFA and PUFA in muscle tissue of those fish were as follows: herring > bream ≈ perch; bream > herring > perch and perch > herring > bream, respectively. In turn, Kołakowska et al. [63] observed that these groups in muscle lipids of rainbow trout, carp and flounder was as follows: flounder > rainbow trout > carp (SFA); carp > flounder > rainbow trout (MUFA) and rainbow trout > flounder > carp (PUFA). The results observed by Polak-Juszczak and Komar-Szymczak [62] and Kołakowska et al. [63] are in not accordance with the results of the present study. Similarly, the previously findings reported by Łuczyńska et al. [60] did not confirm the regularity of these fatty acids in muscles of carp, rainbow trout and bream from Polish market.

According to Ehsani et al. [64], monounsaturated fatty acids (MUFA) in fillets of rainbow trout were the highest, followed by polyunsaturated (PUFA) and saturated fatty acids (SFA). These results are not consistent with those of the present study, because the content of PUFA in muscles of the examined fish was higher than the content of MUFA (Table 2). Karaçali et al. [65] showed that MUFA in muscles of carp (Turkey), independent of seasonal variations, was at the higher amount (45.67–50.17%) than SFA (25.29–28.13%) and PUFA (17.87–26.73%). These findings are not in agreement with those reported by Donmez [66] for muscles carp living in Porsuk Dam, Turkey (SFA > MUFA ≈ PUFA). However, Ćirković et al. [67] found that muscles of carp in raised in poly-culture (Serbia) had more PUFA than SFA and MUFA. According to these authors, nutrient composition, varies widely among fish species, especially the profile of fatty acids related to their consumption habits (herbivorous, omnivorous and carnivorous). Polak-Juszczak and Komar-Szymczak [62] observed that muscle tissue of bream had more MUFA than PUFA and SFA, whereas the muscle of herring had more PUFA than MUFA and SFA, and these groups in muscles of perch were as follows PUFA > SFA > MUFA. The results of authors are close only to those for perch examined. Stancheva et al. [68] noted that carp from the Danube River contained higher levels of n-3 PUFA in comparison with n-6 PUFA. These results are in good agreement with the data from the present study on the same fish species.

Ouraji et al. [69] and Stancheva et al. [68] reported that higher values of AI and TI (>1.0) are detrimental to human health. The value of these parameters in muscles of carp, both studied by Stancheva et al. [68] (0.65 and 0.36, respectively) and in the present study, were lower than 1.0. The FLQ value (6.84) in the muscle tissue of carp reported by Stancheva et al. [68] was lower than those noted in present study. Indices such as AI and TI in fillets of rainbow trout with three different average weights ranged from 0.20 to 0.28 and from 0.88 to 1.28, respectively [64]. These values are superior to those for rainbow trout studied in the present study. The same author found that fish weighing about 480 g and 350 g contained lower AI and TI values than the low-weight fish, and that the content of DHA in fillets of rainbow trout decreased with weight. For the examined rainbow trout, DHA decreased with increased their weight, but the correlation was not significant. 

A consumer who eats 232.12 g of weekly portion of fish meat ingests less mercury than the TWI [23], which means that it does not pose any health risks. The THQ of mercury in species examined for adult was also less than 1. Addo-Bediako et al. [70] reported that THQ < 1 suggests that adverse health effects are unlikely, whereas THQ > 1 suggests a high probability of adverse health effects. This shows that the fish from Polish market are safe for consumers. Although the greatest contribution of harmful exposure to mercury for humans is through eating fish, we should comprehensively address the subject and take into account the multiple sources of exposure. For example mercury content in fresh fruit and vegetables from independent agrarian production ranged between 0.0011 and 0.0039 mg/kg (Poland) [71]. However, vegetable products, products for infants and children and wheat cereal products contained 0.001–0.008 mg/kg. These values did not affect health and were generally below the levels set forth in food legislation (0.01 mg/kg) [72]. The results observed by Duma et al. [73] also showed that the analysed products collected each year during 2002–2010 from selected farms in the Podkarpackie Province (Poland) did not present a risk to human health because the content of mercury (<0.001–0.003 mg/kg) in the tested products (milk, pigs, pork) did not exceed the maximum admissible values. Mercury, which has been acknowledged as a serious human toxin, was absent in the fruits and vegetable in Lagos state (Nigeria) [74]. Mercury content had no hazardous effect on human health and environment pollution in Shahre-Ray regions (Iran) because the mean concentrations of mercury in five leafy vegetables was 0.027 mg/kg dry weight [75]. The same authors found that mercury also had a low concentration in soil and water as compared with WHO/FDA references. The concentration of Hg in various vegetables (roots, stems, leafy, fruits, cereals and legumes) grown in four major industrial and urban cities (Tabouk, Riyadh, Damamm and Jazan) in Saudi Arabia was not detected in selected legume species from the northern district while it was at low levels in leafy vegetables (except parsley, which recorded the maximum mercury value (0.048 mg/kg dry weight) [76]. According to these data, fish are more likely to be exposed to the toxic effects of mercury and contain more than other raw materials and food products.

## 5. Conclusions

The examined fish are better dietary sources of n-3 PUFA. Despite this, n-3/n-6 ratio in marine fish was higher than other fish examined, which is associated with a small amount of n-6 PUFA in lipid muscles of this group. Furthermore, all fish species had more hypercholesterolemic fatty acids relative to hypocholesterolemic fatty acids and may be an important dietetic fish food from a cardiovascular disease point of view. The dietetic quality indices of lipids (index of flesh-lipid quality, atherogenicity and thrombogenicity) was no more than 1.0 which according to the data literature is detrimental to human health. In conclusion, the fish examined did not exceed the maximum acceptable level of mercury and were a beneficial source of PUFA, especially n-3 PUFA may be recommended for human health consumption, especially since the Target Hazard Quotient (THQ < 1) showed there is a non-carcinogenic health risk to the consumer.

## Figures and Tables

**Table 1:** Mercury and total lipids content (mean ± SD, range), and linear correlation coefficients (r) between body weight or total length and content of mercury in muscles of fish.

Species	Body Weight (g)	Total Length (cm)	Total Lipids (%)	Hg (mg/kg Wet Weight)	Hg (mg/100g Fat)	Body Weight (r)	*p*	Total Length (r)	*p*
Bream *Abramis brama* L. *n* = 6	207.7 ± 20.5 192.0–248.0	26.7 ± 1.4 25.5–29.5	0.115 ± 0.050 d 0.080–0.200	0.016 ± 0.009 c 0.006–0.027	1.467 ± 0.904 0.550–3.000	0.580	0.227	0.476	0.340
Perch *Perca fluviatilis* L. *n* = 5	561.2 ± 155.1 296.0–704.0	32.7 ± 2.6 28.0–34.5	0.352 ± 0.134 d 0.230–0.560	0.138 ± 0.111 a 0.078–0.336	4.160 ± 3.262 1.875–9.882	0.601	0.283	0.459	0.437
Ide *Leuciscus idus* L. *n* = 6	950.0 ± 179.3 742.0–1266.0	40.6 ± 3.1 36.0–43.8	0.802 ± 0.378 cd 0.400–1.400	0.109 ± 0.050 a 0.046–0.161	1.716 ± 1.283 0.357–4.025	0.787	0.063	0.183	0.729
Carp *Cyprinus carpio* L. *n* = 5	1197.2 ± 198.8 938.0–1432.0	34.6 ± 0.5 34.0–35.2	0.684 ± 0.494 d 0.210–1.470	0.006 ± 0.002 d 0.004–0.009	0.158 ± 0.161 0.041–0.429	0.878	0.050	0.683	0.204
Rainbow trout *Oncorhynchus mykiss* Walb. *n* = 6	202.7 ± 33.8 158.0–238.0	25.3 ± 1.0 24.0–26.2	2.055 ± 0.368 b 1.470–2.460	0.015 ± 0.001 c 0.013–0.016	0.073 ± 0.010 0.063–0.088	0.429	0.396	0.336	0.514
Flounder *Platichthys flesus* L. *n* = 12	274.1 ± 64.9 195.0–369.0	28.6 ± 1.6 27.0–31.7	1.770 ± 0.719 bc 0.850–2.340	0.056 ± 0.020 b 0.028–0.084	0.354 ± 0.164 0.221–0.671	0.925	0.008	0.600	0.208
Herring *Clupea harengus* L. *n* = 12	182.9 ± 30.2 142.0–227.0	26.1 ± 0.9 24.9–27.6	11.487 ± 1.834 a 9.620–14.560	0.021 ± 0.012 c 0.007–0.039	0.018 ± 0.009 0.006–0.033	0.982	0.0005	0.794	0.059

*n*—Number of fish; SD—standard deviation; a, b, c, d—significant differences at *p* ≤ 0.05. The same letter indicates a lack of significant differences between the muscles fish species (*p* > 0.05); *p*—significance levels for the correlation between the content of mercury in muscles of fish and their body weight or total length.

**Table 2:** Lipid content (%) and fatty acids composition (% of total fatty acids) in muscles of different fish species.

Fatty Acids	Bream		Perch		Ide		Carp		Rainbow Trout		Flounder		Herring	
	$\bar{x}$	SD	$\bar{x}$	SD	$\bar{x}$	SD	$\bar{x}$	SD	$\bar{x}$	SD	$\bar{x}$	SD	$\bar{x}$	SD
*n*	6		5		6		5		6		12		12	
fat	0.12	0.05	0.35	0.13	0.80	0.38	0.68	0.49	2.06	0.37	1.77	0.72	11.49	1.83
C12:0	0.11 ab	0.04	0.07 cd	0.00	0.11 ab	0.04	0.10 bc	0.03	0.05 d	0.01	0.08 bcdd	0.02	0.14 a	0.02
C14:0	0.82 e	0.17	0.97 de	0.13	1.47 cde	0.60	1.58 cd	0.39	1.93 c	0.11	3.51 b	0.26	9.77 a	1.13
C15:0	0.91 a	0.19	0.47 c	0.05	0.59 bc	0.19	0.45 c	0.05	0.18 d	0.01	0.72 b	0.10	0.49 c	0.05
C16:0	21.27 ab	1.51	21.97 a	1.80	19.75 b	2.29	19.39 b	1.07	16.28 c	0.61	16.74 c	1.18	13.00 d	1.54
C17:0	1.63 b	0.15	0.68 c	0.05	0.61 cd	0.15	0.66 c	0.11	3.43 a	0.38	0.40 de	0.06	0.20 e	0.03
C18:0	7.82 a	0.46	5.74 bc	0.49	5.19 c	1.49	6.04 b	0.48	0.18 f	0.01	2.88 d	0.43	0.99 e	0.07
C20:0	0.17 cd	0.04	0.11 d	0.02	0.15 d	0.06	0.27 bc	0.04	4.06 a	0.20	0.09 d	0.02	0.33 b	0.05
C22:0	0.20 a	0.05	0.01 c	0.00	<0.01 c	0.00	<0.01 c	0.00	0.16 b	0.02	<0.01 c	0.00	<0.01 c	0.00
ΣSFA	32.94 a	2.18	30.01 b	2.25	27.86 bc	2.57	28.49 bc	1.14	26.27 cd	0.67	24.42 d	1.62	24.92 d	2.64
C14:1	0.01 c	0.00	0.05 b	0.02	0.06 ab	0.04	0.06 ab	0.03	0.02 c	0.00	0.08 a	0.02	0.08 a	0.01
C16:1	2.84 d	0.55	5.46 c	2.11	7.65 b	0.88	10.49 a	1.22	0.12 e	0.01	21.17 a	3.25	4.60 cd	0.39
C17:1	0.80 cd	0.13	0.61 de	0.10	0.88 bc	0.32	1.09 ab	0.25	0.31 f	0.01	1.17 a	0.21	0.54 ef	0.09
C18:1	9.73 e	1.06	12.20 d	3.36	17.21 c	1.03	22.74 b	2.70	27.78 a	1.72	16.35 c	1.69	7.15 f	0.74
C20:1(n-7)	0.25 b	0.10	0.12 b	0.01	0.26 b	0.06	0.23 b	0.04	0.08 b	0.01	3.45 a	1.02	0.17 b	0.02
C20:1(n-9)	0.18 e	0.11	0.33 de	0.10	0.65 cde	0.08	1.38	0.37 b	0.84 bcd	0.09	1.05 bc	0.25	14.99 a	1.03
C20:1(n-11)	0.33 c	0.12	<0.01 d	0.00	0.24 cd	0.14	0.72 b	0.08	<0.01 d	0.00	1.80 a	0.44	<0.01 d	0.00
C22:1(n-9)	<0.01 c	0.00	<0.01 c	0.00	<0.01 c	0.00	<0.01 c	0.00	<0.01 c	0.00	0.16 b	0.13	0.92 a	0.14
C22:1(n-11)	<0.01 b	0.00	<0.01 b	0.00	<0.01 b	0.00	<0.01 b	0.00	0.22 b	0.13	0.17 b	0.17	26.08 a	2.59
ΣMUFA	14.13 f	1.51	18.77 e	5.02	26.94 d	1.56	36.50 c	3.47	29.37 d	1.80	45.40 b	4.19	54.54 a	2.90
C18:2(n-6)	3.06cd	0.27	2.35 d	0.99	3.34 c	0.62	6.41 b	0.83	12.47 a	0.60	1.26 e	0.14	1.49 e	0.23
C18:3γ-lin (n-6)	0.39 b	0.04	0.33 b	0.06	0.24 c	0.04	0.49 a	0.08	0.40 b	0.08	0.17 cd	0.04	0.11 d	0.01
C20:2(n-6)	0.83 a	0.10	0.27 c	0.04	0.82 a	0.19	0.77 a	0.09	0.50 b	0.04	0.43 b	0.08	0.20 c	0.02
C20:3(n-6)	0.39 b	0.09	0.36 b	0.07	0.45 b	0.11	1.01 a	0.10	0.44 b	0.04	0.09 c	0.03	<0.01 c	0.00
C20:4(n-6)	9.47 a	0.64	7.67 b	0.83	7.39 b	1.92	5.53 c	0.68	0.97 e	0.10	2.82 d	0.40	0.35 e	0.06
C22:5(n-6)	1.33 b	0.36	1.61 a	0.12	1.03 c	0.12	0.89 c	0.19	0.25 de	0.03	0.36 d	0.06	0.10 e	0.03
C18:3(n-3)	2.33 bc	0.56	1.79 cd	0.44	2.74 b	1.22	2.07 bc	0.51	7.67 a	0.17	0.63 e	0.11	1.15 de	0.20
C18:4(n-3)	0.12 c	0.05	0.26 c	0.05	0.27 c	0.27	0.27 c	0.12	1.39 b	0.24	1.16 b	0.21	2.87 a	0.56
C20:3(n-3)	0.94 a	0.16	0.31 b	0.07	0.94 a	0.41	0.26 b	0.05	0.30 b	0.03	0.18 b	0.04	0.09 b	0.02
C20:4(n-3)	0.72 ab	0.15	0.52 b	0.11	0.92 a	0.68	0.63 ab	0.15	0.70 ab	0.09	0.35 b	0.05	0.51 b	0.08
C20:5(n-3) EPA	10.51 b	1.83	6.39 cd	0.52	7.53 c	0.70	6.19 cd	1.17	5.00 d	0.44	12.58 a	1.55	5.40 d	0.63
C22:5(n-3)	3.21 a	0.54	2.52 a	0.53	2.77 a	1.35	2.69 a	0.43	1.30 b	0.11	2.48 a	0.35	0.66 b	0.15
C22:6(n-3)DHA	19.63 b	1.50	26.82 a	1.51	16.79 c	2.02	7.80 e	1.17	12.97 d	2.13	7.67 e	0.87	7.61 e	0.87
ΣPUFA	52.93 a	2.63	51.21 a	2.92	45.20 b	3.16	35.01 c	3.35	44.36 b	2.02	30.18 d	2.92	20.54 e	1.27
n-3/n-6	2.43 cd	0.22	3.07 c	0.12	2.56 cd	0.84	1.32 e	0.13	1.96 d	0.22	4.93 b	0.59	8.15 a	0.73
Σn-6 PUFA	15.48 a	0.95	12.60 b	0.91	13.26 b	2.56	15.10 a	0.84	15.03 a	0.62	5.13 c	0.63	2.26 d	0.23
Σn-3 PUFA	37.46 a	2.45	38.62 a	2.10	31.94 b	4.83	19.91 d	2.66	29.33 b	2.43	25.05 c	2.66	18.28 d	1.16
Σn-3 HUFA	35.00 a	2.27	36.57 a	2.55	28.94 b	3.90	17.57 d	2.60	20.26 cd	2.55	23.26 c	2.56	14.27 e	0.86
OFA	22.21 ab ab	1.52	23.01 a	1.91	21.33 ab	1.83	21.07 ab	1.18	18.26 c	0.62	20.34 bc	1.19	22.92 a	2.56
DFA	74.88 c	1.80	75.73 bc	1.93	77.33 ab	1.61	77.55 ab	1.18	73.91 c	0.66	78.46 a	1.25	76.07 bc	2.60
AI	0.37 bc	0.04	0.37 bc	0.04	0.36 bc	0.02	0.36 bc	0.03	0.33 c	0.01	0.41 b	0.03	0.70 a	0.10
TI	0.23 bc	0.03	0.21 c	0.01	0.22 bc	0.05	0.31 a	0.03	0.16 d	0.01	0.22 c	0.02	0.26 b	0.04
FLQ	30.14	1.82	33.22	1.84	24.32	2.47	13.99	2.15	17.97	2.46	20.25	2.30	13.01	0.77

*n*—Number of fish; SD—standard deviation; a, b, c, d, e, f—significant differences (*p* ≤ 0.05). The same letter (in rows) indicates the absence of significant differences (*p* > 0.05). ΣSFA (saturated fatty acid), ΣMUFA (monounsaturated fatty acid). The same letter (in rows) indicates the absence of significant differences h (*p* > 0.05). EPA-eicosapentaenoic acid (C20:5). DHA-docosahexaenoic (C22:6). Σn-6 PUFA (polyunsaturated fatty acid). Σn-3 PUFA (polyunsaturated fatty acid). Σn-3 HUFA (highly unsaturated fatty acid) contains C20:3, C20:4, C20:5 EPA, C22:5 and C22:6 DHA. AI—index of atherogenicity, TI—index of thrombogenicity, FLQ—flesh-lipid quality, OFA—hypocholesterolemic fatty acids, DFA—hypercholesterolemic fatty acids; “<” indicates that the contents are less than 0.01%.

**Table 3:** Linear correlation coefficients (r) between fatty acids content in muscles of fish and body weight or total length.

Fatty Acids	Body Weight (r)	*p*	Total Length (r)	*p*	Body Weight (r)	*p*	Total Length (r)	*p*	Body Weight (r)	*p*	Total Length (r)	*p*	Body Weight (r)	*p*	Total Length (r)	*p*
Species	Bream				Perch				Ide				Carp				
C12:0	−0.307	ns	0.005	ns	0.125	ns	0.173	ns	−0.320	ns	−0.692	ns	0.460	ns	0.557	ns
C14:0	−0.370	ns	−0.071	ns	−0.485	ns	−0.407	ns	0.570	ns	0.292	ns	0.867	ns	0.544	ns
C16:0	−0.147	ns	−0.092	ns	−0.299	ns	−0.136	ns	−0.504	ns	−0.672	ns	0.050	ns	0.029	ns
C18:0	0.153	ns	0.225	ns	−0.783	ns	−0.678	ns	−0.200	ns	−0.383	ns	−0.239	ns	0.155	ns
C18:2(n-6)	−0.735	ns	−0.845	0.034	0.908	0.033	0.927	0.024	−0.182	ns	−0.136	ns	0.883	0.047	0.603	ns
C20:4(n-6)	0.361	ns	0.100	ns	−0.806	ns	−0.679	ns	−0.743	ns	−0.632	ns	−0.551	ns	0.114	ns
C18:3(n-3)	0.086	ns	0.243	ns	0.865	ns	0.806	ns	0.582	ns	0.553	ns	0.701	ns	0.490	ns
C20:3(n-3)	0.224	ns	0.439	ns	0.717	ns	0.708	ns	0.442	ns	0.521	ns	0.266	ns	0.414	ns
C20:4(n-3)	0.112	ns	0.292	ns	0.523	ns	0.559	ns	0.510	ns	0.585	ns	0.498	ns	0.569	ns
C20:5(n-3)	0.816	0.047	0.724	ns	−0.930	0.022	−0.846	ns	−0.189	ns	0.140	ns	0.020	ns	0.578	ns
C22:5(n-3)	0.722	ns	0.571	ns	−0.916	0.029	−0.876	ns	0.320	ns	0.621	ns	−0.417	ns	0.265	ns
C22:6(n-3)	−0.216	ns	−0.203	ns	−0.254	ns	−0.104	ns	0.124	ns	0.772	ns	−0.336	ns	0.311	ns
ΣSFA	−0.206	ns	−0.088	ns	−0.472	ns	−0.313	ns	−0.410	ns	−0.759	ns	0.348	ns	0.450	ns
ΣMUFA	−0.598	ns	−0.564	ns	0.363	ns	0.211	ns	0.557	ns	−0.345	ns	−0.198	ns	−0.781	ns
ΣPUFA	0.513	ns	0.396	ns	−0.261	ns	−0.122	ns	0.059	ns	0.835	0.038	0.087	ns	0.657	ns
n-3/n-6	0.734	ns	0.876	0.022	−0.687	ns	−0.788	ns	0.563	ns	0.680	ns	−0.229	ns	0.370	ns
Σn-6 PUFA	−0.322	ns	−0.572	ns	0.064	ns	0.214	ns	−0.685	ns	−0.576	ns	0.359	ns	0.756	ns
Σn-3 PUFA	0.675	ns	0.646	ns	−0.391	ns	−0.262	ns	0.416	ns	0.821	0.045	−0.003	ns	0.590	ns
Σn-3 HUFA	0.709	ns	0.635	ns	−0.546	ns	−0.417	ns	0.297	ns	0.836	0.038	−0.175	ns	0.483	ns
OFA	−0.196	ns	−0.100	ns	−0.317	ns	−0.157	ns	−0.454	ns	−0.756	ns	0.348	ns	0.224	ns
DFA	0.289	ns	0.164	ns	0.351	ns	0.193	ns	0.457	ns	0.837	0.038	−0.431	ns	−0.371	ns
AI	−0.279	ns	−0.114	ns	−0.379	ns	−0.226	ns	−0.125	ns	−0.890	0.018	0.707	ns	0.496	ns
TI	−0.375	ns	−0.298	ns	−0.417	ns	−0.257	ns	−0.458	ns	−0.812	0.050	0.145	ns	−0.301	ns
FLQ	0.644	ns	0.562	ns	−0.468	ns	−0.322	ns	0.052	ns	0.687	ns	−0.171	ns	0.482	ns
Species	Rainbow trout				Flounder				Herring								
C12:0	−0.280	ns	−0.396	ns	0.247	ns	−0.078	ns	0.088	ns	−0.335	ns				
C14:0	0.735	ns	0.504	ns	0.888	0.018	0.632	ns	−0.038	ns	−0.212	ns				
C16:0	−0.170	ns	−0.418	ns	0.109	ns	−0.410	ns	−0.258	ns	−0.177	ns				
C18:0	0.087	ns	−0.122	ns	0.410	ns	0.015	ns	0.164	ns	0.280	ns				
C18:2(n-6)	0.490	ns	0.533	ns	0.251	ns	−0.166	ns	−0.409	ns	−0.056	ns				
C20:4(n-6)	0.119	ns	−0.106	ns	0.406	ns	0.043	ns	0.118	ns	0.032	ns				
C18:3(n-3)	−0.319	ns	0.024	ns	−0.195	ns	−0.142	ns	−0.287	ns	−0.190	ns				
C20:3(n-3)	−0.135	ns	−0.073	ns	−0.155	ns	−0.181	ns	−0.379	ns	−0.265	ns				
C20:4(n-3)	0.119	ns	−0.106	ns	−0.149	ns	0.348	ns	−0.669	ns	−0.447	ns				
C20:5(n-3)	−0.805	ns	−0.661	ns	0.120	ns	0.065	ns	0.343	ns	0.045	ns				
C22:5(n-3)	−0.713	ns	−0.437	ns	−0.295	ns	−0.031	ns	0.157	ns	0.095	ns				
C22:6(n-3)	−0.793	ns	−0.719	ns	0.043	ns	−0.350	ns	−0.461	ns	−0.309	ns				
ΣSFA	0.436	ns	0.142	ns	0.357	ns	−0.158	ns	−0.156	ns	−0.177	ns				
ΣMUFA	0.835	0.039	0.753	ns	−0.191	ns	0.105	ns	0.346	ns	0.341	ns				
ΣPUFA	−0.887	0.018	−0.717	ns	0.076	ns	−0.063	ns	−0.465	ns	−0.409	ns				
n-3/n-6	−0.762	ns	−0.697	ns	−0.325	ns	−0.016	ns	0.100	ns	−0.341	ns				
Σn-6 PUFA	0.425	ns	0.491	ns	0.339	ns	−0.042	ns	−0.448	ns	−0.042	ns				
Σn-3 PUFA	−0.845	0.034	−0.721	ns	0.003	ns	−0.060	ns	−0.422	ns	−0.440	ns				
Σn-3 HUFA	−0.831	0.040	−0.740	ns	0.042	ns	−0.079	ns	−0.259	ns	−0.311	ns				
OFA	−0.037	ns	−0.323	ns	0.307	ns	−0.266	ns	−0.171	ns	−0.202	ns				
DFA	−0.438	ns	−0.144	ns	−0.321	ns	0.210	ns	0.163	ns	0.188	ns				
AI	0.334	ns	0.003	ns	0.618	ns	0.031	ns	−0.098	ns	−0.192	ns				
TI	0.741	ns	0.468	ns	0.370	ns	−0.152	ns	−0.065	ns	−0.057	ns				
FLQ	−0.830	0.041	−0.740	ns	0.097	ns	−0.088	ns	−0.242	ns	−0.314	ns				

*p*—Significance levels for the correlation between the content of fatty acids in muscles of fish and their body weight or total length, ns—non-significant correlation. ΣSFA (saturated fatty acid), ΣMUFA (monounsaturated fatty acid), Σn-6 PUFA (polyunsaturated fatty acid), Σn-3 PUFA (polyunsaturated fatty acid), Σn-3 HUFA (highly unsaturated fatty acid) contains C20:3, C20:4, C20:5 EPA, C22:5 and C22:6 DHA. AI—index of atherogenicity, TI—index of thrombogenicity, FLQ—flesh-lipid quality, OFA—hypocholesterolemic fatty acids, DFA—hypercholesterolemic fatty acid.

**Table 4:** The Hazard Quotient calculated for mercury content in the muscle tissue of fish.

Species	EDI	EWI	%TWI *	%TWI **	THQ
Bream (*Abramis brama* L.) *n* = 6	0.0086	0.060	1.50	4.611	0.029
Perch (*Perca fluviatilis* L.) *n* = 5	0.0762	0.534	13.34	41.056	0.254
Ide (*Leuciscus idus* L.) *n* = 6	0.0604	0.423	10.57	32.527	0.201
Carp (*Cyprinus carpio* L.) *n* = 5	0.0043	0.024	0.60	1.845	0.011
Rainbow trout (*Oncorhynchus mykiss* Walb.) *n* = 6	0.0081	0.057	1.42	4.363	0.027
Flounder (*Platichthys flesus* L.) *n* = 12	0.0311	0.218	5.45	16.760	0.104
Herring (*Clupea harengus* L.) *n* = 12	0.0116	0.081	2.03	6.248	0.039

*n*—Number of fish; EDI—the estimated daily intake (μg/kg body weight/day); EWI—the estimated weekly intake (μg/kg body weight/weekly); THQ—Target Hazard Quotient; * TWI—Tolerable Weekly Intake for inorganic mercury expressed as mercury = 4 μg/kg body weight, ** TWI for methylmercury expressed as mercury is 1.3 μg/kg body weight.
